# Identifying Predictors of Lung Volume in Pediatric Patients Undergoing Surgery: A STROBE-Compliant Retrospective Cross-Sectional Chest Computed Tomography Study

**DOI:** 10.3390/jcm15062313

**Published:** 2026-03-18

**Authors:** Sou-Hyun Lee, Dong Gun Lim, Sung-Sik Park, Younghoon Jeon, Jinseok Yeo, Hoon Jung, Jiyong Yeom, Chanhyo Choi, Kyung-Hwa Kwak

**Affiliations:** 1 Department of Anesthesiology and Pain Medicine, Kyungpook National University Hospital, Kyungpook National University School of Medicine, Daegu 41944, Republic of Korea; youlion6@gmail.com (S.-H.L.);; 2 Department of Anesthesiology and Pain Medicine, Kyungpook National University Chilgok Hospital, Kyungpook National University School of Medicine, Daegu 41404, Republic of Korea

**Keywords:** computed tomography, linear model, pediatrics, respiratory physiological phenomena, total lung capacity

## Abstract

**Background/Objectives**: Tidal volume is determined by height and sex in adults under mechanical ventilation, and it serves as the foundation for implementing a lung-protective ventilation strategy. In children, tidal volume is often calculated based on actual body weight, without established guidelines regarding the predictors of lung volume. The aim of this study was to identify the key predictors of lung volume in children aged 0–5 years. **Methods**: This retrospective study involved 51 children aged 0–5 years who underwent chest computed tomography (CT) and surgery under general anesthesia between 2014 and 2024. The total lung volume was calculated using three-dimensional segmentation of the CT images. Linear regression models were used to assess predictors, including height, weight, age, sex, and body mass index (BMI). Model performance was evaluated using the adjusted R-squared and Akaike Information Criterion (AIC). Bootstrap validation with 2000 iterations was used to validate model reliability. **Results**: Height was the strongest predictor of lung volume (adjusted R-squared: 0.5621), and it showed a collinearity with age. The final model included age and sex as the covariates. The Bootstrap validation confirmed the model’s reliability. **Conclusions**: Age and sex are key predictors of the CT-derived total lung volume in children aged 0–5 years. Further studies are required to validate these findings. In addition, research is needed to derive and validate a tidal volume equation based on these predictors and assess the influence of this equation on clinical outcomes such as atelectasis, oxygenation, and inflammatory markers in pediatric surgery.

## 1. Introduction

Accurate estimation of lung volume is crucial in pediatric care, as it directly influences the effectiveness of ventilation strategies and reduces the risk of complications such as volutrauma and atelectotrauma in children on mechanical ventilation [1]. In adults, lung volume is primarily determined by sex and height [2]. Lung-protective ventilation strategies based on predicted body weight (PBW), derived from these factors, decrease mortality compared with strategies based on actual body weight in patients with acute respiratory distress syndrome [3]. Additionally, these strategies are associated with lower levels of inflammatory markers [4]. Based on these findings, a low tidal volume ventilation of 6–8 mL/kg of PBW is established as a widely accepted standard in anesthesia practice [5].

No consensus has been established for pediatric patients, in whom tidal volume is commonly set at 7–8 mL/kg of actual body weight [6]. However, reliance on a weight-based approach alone may result in inappropriate ventilation, as it fails to account for other critical covariates, particularly in children whose lung volume is still developing. Thus, there is a need for more comprehensive and predictive modeling approaches.

Although researchers have investigated the determinants of lung volume in children, their findings have been inconsistent, complicating the development of evidence-based guidelines. One study identified age as the primary covariate [7], whereas another suggested that sex, age, and height are key determinants [8]. However, these studies did not perform multivariate analyses to account for multiple factors, and their models were not rigorously validated, limiting their clinical applicability.

As directly measuring tidal volume in pediatric patients is challenging, in this study, we aimed to identify the key predictors of total lung volume in children aged 0–5 years using multivariate analysis to account for various factors. We hypothesized that, as in adults, sex and height are the primary covariates in children.

## 2. Materials and Methods

### 2.1. Study Design and Population

This retrospective cross-sectional study was approved by the Institutional Review Board of Kyungpook National University Hospital (approval number: 2024-08-010; 10 September 2024). Obtaining patient consent was not required owing to the retrospective nature of the study. The study adheres to the STrengthening the Reporting of OBservational studies in Epidemiology (STROBE) guidelines.

### 2.2. Data Collection

Korean children aged 0–5 years who underwent surgery under general anesthesia and had a chest computed tomography (CT) scan between January 2014 and June 2024 were selected from the Clinical Data Warehouse of Kyungpook National University Hospital. Patients with an American Society of Anesthesiologists physical status (ASA-PS) score of 3 or higher, the presence of pulmonary or cardiac lesions on the chest CT reading, or a chest CT taken more than 1 month before the date of surgery were excluded. Data on patient sex, age (months), height (cm), weight (kg), ASA-PS score, and type of surgery, as well as respiratory status at the time of CT acquisition (spontaneous breathing or mechanical ventilation) and use of sedation, were collected from electronic medical records. Age, height, and weight data were obtained from measurements taken on the day of surgery. Non-contrast axial chest CT images were retrieved through INFINITT PACS version G3.

### 2.3. Total Lung Volume Calculation

Total lung volume, from the vocal cords to the lung parenchyma, was calculated using MATLAB R2024a (MathWorks, Inc., Natick, MA, USA) and Image Processing Toolbox. Non-contrast, axial chest CT images were imported using a DICOM browser in Image Processing Toolbox. The intensity windows were adjusted to a Hounsfield unit range of −1200 to 1200. Three-dimensional segmentation and lung volume calculation were performed as reported previously [9]. A detailed description of the MATLAB code and 3D lung volume calculations is presented in Figures S1 and S2.

### 2.4. Statistical Analysis

Continuous variables with a normal distribution (e.g., age, total lung volume) are presented as mean (standard deviation); non-normally distributed continuous variables (e.g., height, weight) as median (Q1, Q3); body mass index (BMI) as median [minimum, maximum]; and categorical variables as count (%).

Simple linear regression analyses were performed for each covariate to identify those associated with total lung volume. Adjusted R-squared values were calculated to evaluate the explanatory power of each variable in predicting total lung volume, using the Curve Fitting Toolbox in MATLAB. The variables analyzed included age, sex, height [8], weight [7], and BMI [10]. Covariate modeling was conducted for linear regression using stepwise forward inclusion, starting with the variable that had the highest adjusted R-squared value from the simple regression analysis, using the fitlm function in the Statistics and Machine Learning Toolbox. Height was entered as height/median height to improve interpretability of the covariate function by scaling the continuous variable to a typical reference value [11].

The final model was selected based on the lowest Akaike Information Criterion (AIC) value [12], which was calculated using the AIC function in the System Identification Toolbox.

Correlation between variables was assessed using correlation coefficients with the corr function in the Statistics and Machine Learning Toolbox [13]. Pairs of variables with correlation coefficients exceeding 0.70 were considered strongly correlated [14]. Multicollinearity was evaluated using the variance inflation factor (VIF) and variance decomposition proportion (VDP). Multicollinearity was considered present when the VIF exceeded 5 and the VDP was greater than 0.8 [15].

When collinearity existed between two variables, only one was retained during model finalization. The final model’s assumptions, including normality (assessed via Q-Q plot and Lilliefors test), homoscedasticity (residual vs. fitted value scatter plot), and independence of residuals (tested with the Durbin–Watson test [16]), were evaluated [17].

Internal validation of the model was performed using a non-parametric bootstrap approach with 2000 iterations. In each iteration, a bootstrap sample was generated using random sampling with replacement from the original dataset. The mean and 95% confidence interval (CI) of the coefficients were calculated from the bootstrap distributions using the percentile method.

All statistical analyses were performed using MATLAB R2024a (MathWorks, Inc., Natick, MA, USA) and SigmaPlot 15 (Systat Software Inc., Palo Alto, CA, USA).

## 3. Results

**Figure 1:**
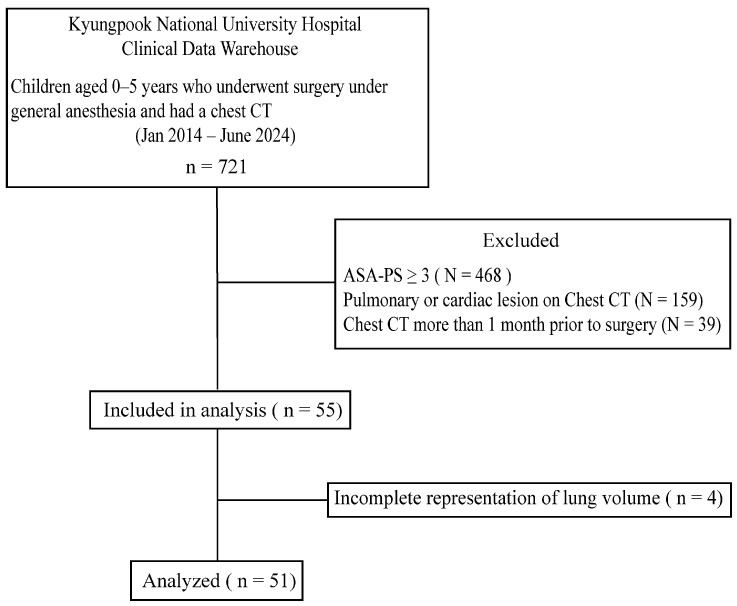
Flow diagram of the study process. ASA-PS, American Society of Anesthesiologists physical status.

Between January 2014 and June 2024, 721 children aged 0–5 years who underwent surgery under general anesthesia and had a chest CT were identified from the Kyungpook National University Hospital Clinical Data Warehouse. After applying the exclusion criteria, 468 children with an ASA-PS score of 3 or higher, 159 children with pulmonary or cardiac lesions on chest CT, and 39 children whose chest CT was performed more than 1 month prior to surgery were excluded. Among the remaining 55 eligible children, 4 were further excluded owing to incomplete representation of the total lung volume; thus, 51 children were included in the final analysis (Figure 1). The characteristics, CT acquisition conditions, and total lung volumes of the study population are presented in Table 1. All children underwent chest CT during spontaneous breathing, and most scans were performed under sedation (36 of 51). Sedative agents included a combination of intravenous midazolam and ketamine or oral chloral hydrate.

**Figure 2:**
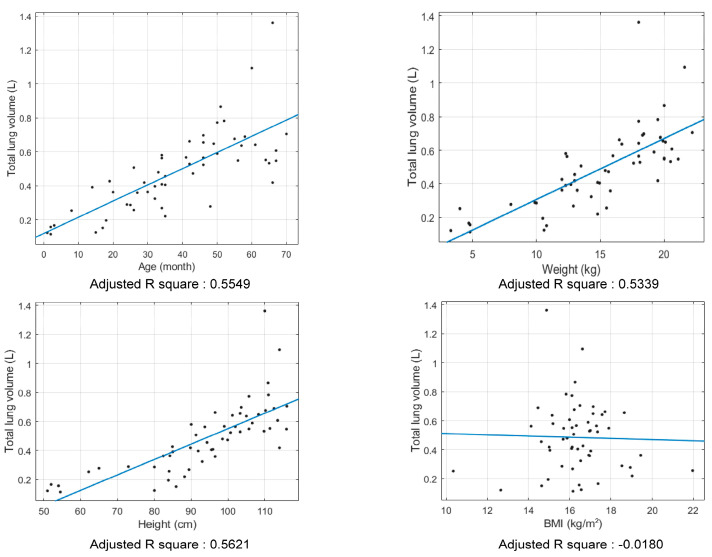
Simple linear regression analyses of total lung volume with covariates. Adjusted R-squared values correspond to the variables on the x-axis in each graph.

In the linear regression analyses, height was the most significant predictor of total lung volume, with an adjusted R-squared value of 0.5621. In contrast, BMI showed a weak explanatory power, with an adjusted R-squared value of −0.0180 (Figure 2).

Stepwise forward linear regression modeling commenced with height as the initial variable. The model that included height, sex, and age demonstrated the lowest AIC value of −51.7301 (Table 2, Model 6). However, because age and height were highly correlated (r = 0.8897, Figure S3) and collinearity was evident (Table 3a,b), models including height + sex and age + sex were compared.

As a result, Model 5, which incorporates age and sex as covariates, was selected as the final model because it achieved a lower AIC (−51.0701 vs. −47.2373) and a higher adjusted R^2^ (0.6168 vs. 0.5892) than the height + sex model (Model 4, Table 2) [18].

The final model satisfied the following key regression assumptions: normality of residuals (Lilliefors test, *p* = 0.084, Q-Q plot in Figure S4), homoscedasticity (Figure S5), and independence of residuals (Durbin–Watson statistic = 1.996, indicating no significant autocorrelation).

Table 4 summarizes the results of the non-parametric bootstrap analysis for the final model of total lung volume. The 95% CIs for the coefficients did not include zero, indicating statistical significance. Additionally, the mean coefficient values from the bootstrap analysis were nearly identical to those of Model 5 in Table 2, indicating accurate parameter estimation.

## 4. Discussion

In this study, age and sex were identified as key covariates of CT-derived total lung volume in pediatric patients. Internal validation of the final models demonstrated reliable parameter estimation. To our knowledge, this is the first study to use multivariate analysis to identify determinants of CT-derived total lung volume in children.

There are two methods to calculate total lung volume, namely, spirometry and CT imaging. In this study, lung volume was measured using CT imaging. This method was chosen because spirometry is particularly challenging to perform accurately in children under 6 years of age owing to limited cooperation, which reduces precision [19]. The reported spirometry success rate among children aged 3–5 years is only 68.4% [20]. Therefore, enrollment was restricted to children aged 0–5 years based on measurement feasibility. Moreover, CT-derived total lung volumes have been shown to closely correlate with those obtained from spirometry, supporting its reliability as an alternative in this population [21].

The mean total lung volume in our patient was 0.50 L, which is similar to the findings of Stein et al. [7]. According to the results presented in Table 1 in Stein et al.’s report, the mean lung volume for children aged 0–5 years was 0.63 L, which is not significantly different from the results of our study. This finding indicates that the MATLAB method that we used is methodologically sound and appropriate.

In our analysis, weight accounted for 53.39% of the variance in the total lung volume (adjusted R-squared value = 0.5339). However, when compared to height, this proportion was low. Height accounted for 56.21% of the variance in CT-derived total lung volume (adjusted R-squared value = 0.5621). The relatively weaker association between weight and total lung volume observed in our study suggests that using actual body weight as the sole basis for tidal volume setting may not be optimal.

Our study showed that BMI is a weak predictor of total lung volume (adjusted R-squared value = −0.0180). A previous study reported a predicted decrease of 3.7% in total lung volume with increasing BMI [10], but this finding was based on pediatric patients with obesity. In contrast, the median BMI in our study population was 16.26, which falls within the normal range. As our study did not include patients with a high BMI, the difference in the findings could be attributed to the absence of patients with obesity in our cohort.

In this study, age and height were found to be highly correlated, and collinearity diagnostics confirmed collinearity between them. A previous study indicated that age and height are major determinants of lung volume, but they did not account for the correlation between these variables [8]. Another study excluded height from the analysis and concluded that age was the primary determinant [7]. Our study addressed this limitation by explicitly accounting for the correlation and collinearity between age and height. Although height was the strongest predictor in the univariate analysis, age captured similar growth-related information and produced a slightly better fit in our study cohort. However, because both are proxies for growth, external validation is needed to determine which predictor demonstrates greater transportability across pediatric populations. From a practical perspective, age in months can be readily calculated, whereas accurate height measurement may be challenging in young children. Therefore, in pediatric patients, tidal volume selection based on age in months rather than height may be more practical and clinically useful.

Our study revealed that age and sex are the primary determinants of CT-derived total lung volume in children, in contrast to adults, in whom height and sex are the major determinants. This may be because, in adults, growth ceases and height no longer changes with age, whereas in children, height increases proportionally with age [22]. However, as our study population consisted of relatively healthy children with an ASA score of 1–2, further research is needed to determine whether age remains a major determinant of lung volume in children with developmental delay.

In general, male individuals are taller than female individuals. However, in our study, there was no significant correlation between sex and height. A previous study in adults has shown that even when their heights are the same, female individuals tend to have smaller lung volumes than male individuals [23]. This difference may be explained by the smaller rib cage dimensions observed in female individuals than in male individuals [23]. Additionally, a study including pediatric and adult populations reported that rib cage volume tends to increase with age in both sexes, with male individuals exhibiting a greater increase in volume during childhood than female individuals [24]. Together, these findings support the observation that sex served as a key determinant of CT-derived total lung volume in our study population.

Our study has certain limitations. First, not all CT images were captured at full inspiration because most children underwent CT under sedation, potentially introducing bias in the measurement of total lung volume. Nonetheless, given the limited cooperation of young children, we believe that our analysis based on CT imaging represents the most appropriate approach for this population. Second, as this is a retrospective cross-sectional study, prospective longitudinal studies are needed to validate our findings. However, such studies pose substantial radiation risks, making them difficult to conduct in pediatric populations. Third, although our model was developed using children aged 0–5 years, it remains unclear whether the observed relationships are consistent across all age subgroups within this range. Therefore, external validation in independent cohorts, including age-stratified analyses, is warranted to confirm the applicability of our findings across pediatric subpopulations. Finally, our study included only relatively healthy Korean children aged 0–5 years with ASA scores of 1–2, which may limit the generalizability of our findings to other populations or age groups, as well as to children with developmental delays, chronic illnesses, or other conditions that could affect lung growth and development. In addition, as all participants were Korean, the findings may not be generalizable to other ethnic groups.

## 5. Conclusions

Our study identified age and sex as key predictors of CT-derived total lung volume in pediatric patients aged 0–5 years under heterogeneous acquisition conditions. Incorporating these variables into ventilatory management strategies may offer a more accurate approach than relying on body weight-based ventilation in pediatric anesthesia. Future research should focus on deriving and externally validating a tidal volume equation based on these predictors using standardized acquisition conditions, as CT-derived lung volume does not necessarily reflect true physiologic total lung capacity. Specifically, studies are needed to determine whether ventilation management strategies based on age and sex, as opposed to those based on actual body weight, can reduce atelectasis, improve oxygenation, and enhance inflammatory marker outcomes in pediatric surgery.

## Figures and Tables

**Table 1:** Patient characteristics, CT acquisition conditions, and total lung volume (N = 51).

Variable	Values
Age (months)	38.96 ± 19.11
Male/Female	29 (57%)/22 (43%)
Height (cm)	96.50 (85.00, 108.30)
Weight (kg)	15.65 (12.30, 19.50)
BMI (kg/m^2^)	16.26 [10.34, 21.97]
Total lung volume (L)	0.50 ± 0.22
ASA-PS I/II	5 (10%)/46(90%)
CT acquisition conditions	
Spontaneous breathing	51 (100%)
Sedation	36 (71%)
Operation	
Chemoport insertion/removal	31 (61%)/2 (4%)
Mass excision	10 (20%)
Other	8 (15%)

Values are presented as number (%), mean ± standard deviation, median (Q1, Q3), or median [minimum, maximum]. BMI = body mass index, ASA-PS = American Society of Anesthesiologists physical status.

**Table 2:** Comparison of linear models for total lung volume estimation using covariates.

	Model 1	Model 2	Model 3	Model 4	Model 5 ^b^	Model 6 ^a^
Covariate	Height (cm)	Height (cm), weight (kg)	Height (cm), age (months)	Height (cm), sex	Age (months), sex	Height (cm), sex, age (months)
Equation	b1 + b2 × (height/96.5)	b1 + b2 × (height/96.5) + b3 × weight	b1 + b2 × (height/96.5) + b3 × months	b1 + b2 × (height/96.5) + b3 (if male)	b1 + b2 × months + b3 (if male)	b1 + b2 × (height/96.5) + b3 (if male) + b4 × months
b1	−0.5115	0.4642	−0.2529	−0.5158	0.0605	−0.1959
b2	0.9982	0.0043	0.0048	0.0895	0.1244	0.1086
b3	-	0.9106	0.5695	0.9789	0.0093	0.0060
b4	-	-	-	-	-	0.4045
Adjusted R^2^	0.5621	0.5542	0.5844	0.5892	0.6168	0.6277
AIC	−44.6806	−42.7409	−46.6102	−47.2373	−51.0701	−51.7301

Linear modeling process began with Model 1. Height was divided by the median value of the study population. AIC = Akaike Information Criterion. ^a^ Initial model selected without considering multicollinearity. ^b^ Final model selected after considering multicollinearity.

**Table 3:** (**a**) Collinearity diagnostics: variance inflation factors, condition indices, and variance decomposition proportions by dimension. (**b**) Collinearity diagnostics: condition indices and variance decomposition proportions by dimension.

(**a**)					
**Variables**			**Variance Inflation Factor**		
Height (cm)			43.75 ^a^		
Age (months)			5.57 ^a^		
Male			1.07		
(**b**)					
**Dimension**	**Eigenvalue**	**Condition Index**	**Variance Decomposition Proportions**		
			**Height (cm)**	**Age (Months)**	**Male**
1	1.93	1	0.0498	0.0496	0.0181
2	0.97	1.41	0.0012	0.0079	0.9209
3	0.10	4.30	0.9490 ^b^	0.9426 ^b^	0.0610

Condition indices were obtained from the eigenvalue. Higher values indicate potential collinearity. Variance decomposition proportion is the proportion of each coefficient’s variance attributable to a given dimension. Variables ^a^ with variance inflation factor greater than 5 correspond to variables ^b^ with variance decomposition proportions exceeding 0.8 at the highest condition index, indicating collinearity between height and age.

**Table 4:** Final model parameter estimates with 95% confidence intervals from bootstrap validation.

Parameter	Mean Coefficient (95% CI)
b1	0.0612 (0.0023–0.1507)
b2	0.1234 (0.0564–0.1969)
b3	0.0093 (0.0070–0.0120)

b1, intercept of the model (baseline value when all other covariates are 0); b2, coefficient representing the effect of age (months); b3, coefficient representing the effect of sex (added if the subject is a male individual). CI = confidence interval.

## Data Availability

The datasets generated and/or analyzed in the current study are available at Zenodo (Supplementary Materials).

## Supplementary Materials

The following supporting information can be downloaded at https://doi.org/10.5281/zenodo.18489398. Figure S1: Detailed MATLAB script for 3D lung volume measurement. Figure S2: Individual 3D lung volume images for the study cohort of 51 patients. Figure S3: Correlation matrix. Correlation coefficients greater than 0.7 indicate a strong correlation. Figure S4: Q-Q plot of residuals. Blue markers represent the residuals, and the dashed red line indicates the expected line under normality. Figure S5: Scatter plot showing residuals (y-axis) against fitted values (x-axis). Blue markers represent the residuals. Residuals and the fitted values are uncorrelated.

## Abbreviations

The following abbreviations are used in this manuscript:

PBW	Predicted Body Weight
CT	Computed Tomography
ASA-PS	American Society of Anesthesiologists Physical Status
BMI	Body Mass Index
AIC	Akaike Information Criterion
VIF	Variance Inflation Factor
VDP	Variance Decomposition Proportion
CI	Confidence Interval

## References

[1] Kalikkot Thekkeveedu R., El-Saie A., Prakash V., Katakam L., Shivanna B. (2022). Ventilation-induced lung injury (VILI) in neonates: Evidence-based concepts and lung-protective strategies. J. Clin. Med..

[2] Crapo R.O., Morris A.H., Clayton P.D., Nixon C.R. (1982). Lung volumes in healthy nonsmoking adults. Bull. Eur. Physiopathol. Respir..

[3] Acute Respiratory Distress Syndrome Network (2000). Ventilation with lower tidal volumes as compared with traditional tidal volumes for acute lung injury and the acute respiratory distress syndrome. N. Engl. J. Med..

[4] Wolthuis E.K., Choi G., Dessing M.C., Bresser P., Lutter R., Dzoljic M., van der Poll T., Vroom M.B., Hollmann M., Schultz M.J. (2008). Mechanical ventilation with lower tidal volumes and positive end-expiratory pressure prevents pulmonary inflammation in patients without preexisting lung injury. Anesthesiology.

[5] Young C.C., Harris E.M., Vacchiano C., Bodnar S., Bukowy B., Elliott R.R.D., Migliarese J., Ragains C., Trethewey B., Woodward A. (2019). Lung-protective ventilation for the surgical patient: International expert panel-based consensus recommendations. Br. J. Anaesth..

[6] Kollisch-Singule M., Ramcharran H., Satalin J., Blair S., Gatto L.A., Andrews P.L., Habashi N.M., Nieman G.F., Bougatef A. (2021). Mechanical ventilation in pediatric and neonatal patients. Front. Physiol..

[7] Stein J.M., Walkup L.L., Brody A.S., Fleck R.J., Woods J.C. (2016). Quantitative CT characterization of pediatric lung development using routine clinical imaging. Pediatr. Radiol..

[8] Barrera C.A., Andronikou S., Tapia I.E., White A.M., Biko D.M., Rapp J.B., Zhu X., Otero H.J. (2021). Normal age-related quantitative CT values in the pediatric lung: From the first breath to adulthood. Clin. Imaging.

[9] MathWorks. https://kr.mathworks.com/help/images/segment-lungs-from-3-d-chest-mri-data.html.

[10] Forno E., Han Y.Y., Mullen J., Celedón J.C. (2018). Overweight, obesity, and lung function in children and adults-A meta-analysis. J. Allergy Clin. Immunol. Pract..

[11] Joerger M. (2012). Covariate pharmacokinetic model building in oncology and its potential clinical relevance. AAPS J..

[12] Yamashita T., Yamashita K., Kamimura R. (2007). A stepwise AIC method for variable selection in linear regression. Commun. Stat. Theory Methods.

[13] Shrestha N. (2020). Detecting multicollinearity in regression analysis. Am. J. Appl. Math. Stat..

[14] Schober P., Boer C., Schwarte L.A. (2018). Correlation coefficients: Appropriate use and interpretation. Anesth. Analg..

[15] Kim J.H. (2019). Multicollinearity and misleading statistical results. Korean J. Anesthesiol..

[16] Barker L.E., Shaw K.M. (2015). Best (but oft-forgotten) practices: Checking assumptions concerning regression residuals. Am. J. Clin. Nutr..

[17] Kim J., Kim D.H., Kwak S.G. (2024). Comprehensive guidelines for appropriate statistical analysis methods in research. Korean J. Anesthesiol..

[18] Portet S. (2020). A primer on model selection using the Akaike Information Criterion. Infect. Dis. Model..

[19] Jat K.R., Agarwal S. (2023). Lung function tests in infants and children. Indian J. Pediatr..

[20] Chaya S., Zar H.J., Gray D.M. (2022). Lung function in preschool children in low and middle income countries: An under-represented potential tool to strengthen child health. Front. Pediatr..

[21] Olsen H.J.B., Mortensen J. (2024). Comparison of lung volumes measured with computed tomography and whole-body plethysmography—A systematic review. Eur. Clin. Respir. J..

[22] Cole T.J., Mori H. (2018). Fifty years of child height and weight in Japan and South Korea: Contrasting secular trend patterns analyzed by SITAR. Am. J. Hum. Biol..

[23] Bellemare F., Jeanneret A., Couture J. (2003). Sex differences in thoracic dimensions and configuration. Am. J. Respir. Crit. Care Med..

[24] Weaver A.A., Schoell S.L., Stitzel J.D. (2014). Morphometric analysis of variation in the ribs with age and sex. J. Anat..

